# Development, evaluation and validation of a new instrument for measurement quality of life in the parents of children with chronic disease

**DOI:** 10.1186/1477-7525-8-151

**Published:** 2010-12-23

**Authors:** Małgorzata Farnik, Grzegorz Brożek, Władysław Pierzchała, Jan E Zejda, Michał Skrzypek, Łukasz Walczak

**Affiliations:** 1 Department of Pneumonology, Medical University of Silesia, Katowice, Poland; 2 Department of Epidemiology, Medical University of Silesia, Katowice, Poland; 3 Department of Biostatistics, Faculty of Public Health, Medical University of Silesia, Bytom, Poland; 4 Information Technology Center, Medical University of Silesia, Katowice, Poland

## Abstract

**Background:**

Childhood chronic disease may affect patients' and their family's functioning. Particularly parents, who play an important role in cooperation between patient and health care professionals, report impaired health - related quality of life (HRQOL). The aim of this study was development, evaluation and validation of a new instrument: Quality of Life in a Child's Chronic Disease Questionnaire (QLCCDQ). The questionnaire is addressed to parents of children with a chronic disease.

**Methods:**

Study design included semi structured interview and qualitative study, which allowed to identify most troublesome problems. Following the results the questionnaire was developed, which consists of 15 questions and covers domains - emotions, patients -perceived symptoms, roles limitations. An observational study involving parents of asthma and diabetes children was conducted to assess the psychometric characteristics of the measure. Psychometric testing was based on the reliability of defined subscales, construct validity, reproducibility assessment, as well as comparison between stable/unstable disease stages and parents of healthy children.

**Results:**

Most troublesome concerns for parents of child with chronic disease included emotional distress and feeling depressed due to child's disease, avoiding social interactions due to child's disease or symptoms. 98 parents of children with asthma or insulin - depended diabetes participated in the psychometric testing of QLCCDQ. Internal consistency reliability for the defined subscales ranged between 0.77 and 0.93. Reproducibility based on the weighted kappa coefficients showed expected level of agreement and was almost perfect in case of 8 questions, substantial for 5 questions and moderate for 2 questions. QLCCDQ demonstrated very good construct validity - all subscales showed statistically significant correlations ranging from 0.4 to 0.9. QLCCDQ scores differed significantly by clinical status - parents of children qualified as stable presented higher scores in most subscales in comparison to parents of children with unstable disease.

**Conclusions:**

The QLCCDQ shows good internal consistency, test-retest reliability, and construct validity. The questionnaire may be useful in helping to understand the impact of chronic child's disease on parental perception of health outcomes.

## Background

An increasing interest of the impact of disease on everyday functioning is leading to the development and implementation of health - related quality of life (HRQOL) measures in many studies. Chronic disease may affect not only patients' functioning, but other family members as well. Childhood chronic disease involves all family members, particularly parents, who play an important role in the cooperation between patients and health care professionals. Higher level of psychological distress in parents of children with chronic disease has been reported in several studies [1-5]. Parents experience limitations in regular daily activities and anxiety due to the child's disease [6]. Clinical experience shows that caregiver HRQOL and perception of child's symptoms are important in the diagnosis and control of established asthma [7]. The perspective of both the child and the parent is important in assessing treatment outcomes and planning support strategies. Pediatrics is unique among medical specialties especially because of the presence of parents when health care is provided for the child. This requires a family-oriented approach to care [8].

HRQOL assessment in childhood chronic disease could either be measured as parent-proxy measures of the child's quality of life or as self-reported parental quality of life. Several studies show that parents are reliable at reporting symptoms and physical function, but less reliable at reporting cognitive and emotional well-being of the child [9].

HRQOL of parents could be assessed by a wide range of general status questionnaires such as the Medical Outcomes Survey Short Form 36 (SF 36) [10], EuroQol [11], Health Utility Index [12], and the Quality of Well being Scale [13]. However, these measures do not to allow the assessment of the burden of the child's disease on the parent's HRQOL. Available general questionnaire proposed for caregivers - Pediatric Inventory for Parents [14,15] covers different aspects of parental HRQOL, but no roles function, the questionnaire requires psychometric examination. Another questionnaire which could be used in wide range of clinical conditions is the Impact on Family Function Scale [16]. This measure focuses on impact of the child's disease on family and covers four domains: financial family/social, mastery, personal - strain.

Most of the existing validated measures are specific - questionnaires, dedicated to parents of children with a particular disease and are not recommended as a measure of HRQOL assessment for other clinical conditions. HRQOL of parents of children with diabetes could be assessed by Well-being and Satisfaction of Caregiver's of Children with Diabetes Questionnaire (WE-CARE) [17], Parents Diabetes Quality of Life questionnaire (PDQOL) [5]. The WE-CARE questionnaire is validated measure which covers parents' psychosocial well-being, satisfaction with treatment, ease of insulin use, and treatment acceptance. The PDQOL assesses parental life satisfaction, impact of child's diabetes and disease related worries. Another example of disease specific measure is Paediatric Asthma Caregivers' Quality of Life Questionnaire (PACQLQ), covering three domains: symptoms, emotional function and activity limitation [18]. PACQLQ is 13-items self-administered questionnaire, which was validated and includes emotional and physical context of impairment. Another example of a specific measure to assess parental HRQOL is the questionnaire developed for parents of children with recurrent ear, nose and throat infections [19], which includes domains of emotional health and daily disturbances (PAR-ENT-QoL).

The proposed questionnaire, the Quality of Life in the Child's Chronic Disease Questionnaire (QLCCDQ) deals with daily problems and limitations that concern parents of children with chronic disease. Compared to existing HRQOL measures in parents - the newly developed parental HRQOL assessment and symptom proxy measure could be implemented in different clinical conditions and covers both impact of disease on family and psychosocial aspects of parental functioning. Parental HRQOL assessment would be helpful to optimize clinical judgment as well as to improve clinical decisions. Childhood diabetes and asthma are examples of chronic disease which require continuous monitoring and treatment and were chosen for questionnaire development and validation. The purpose of this paper is to describe the development of the QLCCDQ and report on the assessment of its validity and reliability.

## Methods

The QLCCDQ is a self - report measure of the parent's HRQOL. Only the symptoms subscale is a proxy measure of the parent's HRQOL. The QLCCDQ consists of 15 questions and covers: emotions (4 questions); patient-perceived symptoms (3 questions); and role limitations (8 questions) including social (3 questions), occupational (3 questions), and family roles (2 questions). The QLCCDQ is a self - administered questionnaire, based on a 7 point Likert scale from 1 (most bothered or limited) to 7 (not bothered or limited). The responses concern parental perceptions of the child and their condition over a previous two-week period. Examples of questions representing each domain of QLCCDQ have been demonstrated in Table 1. Some of items were adopted from the SF-36, which is used extensively in a wide range of medical conditions. The scale is based on 7-point scale, which was previously used in other validated measures as PACQLQ. As well as some questions (for example activity limitations) were similar to PAQLQ and mini Asthma Quality Of Life Questionnaire.

**Table 1:** QLCCDQ - examples of questions

Domains								
Symptoms								
	Did the child show any worrying symptoms?							
		All the time	Most of the time	Often	Sometime	Seldom	Very seldom	Never

Emotions								
	Have you experienced anxiety because of your child's health problems?							
		All the time	Most of the time	Often	Sometimes	Seldom	Very seldom	Never

Role functioning								
Social								
	
	Please indicate how much you have been limited by your child's disease in listed activities in past two weeks - Social activities (going to church, cinema, visiting friends)							
		Extremely limited	Very limited	Quite limited	Moderately limited	Somewhat limited	Hardly limited	Not limited
Occupational								
	
	Please indicate how much you have been limited by your child's disease in work related activities in past two weeks							
		Extremely limited	Very limited	Quite limited	Moderately limited	Somewhat limited	Hardly limited	Not limited
Family								
	
	Do you struggle to find time to spend with other family members (spouse, another child) because of your child' disease?							
		Extremely limited	Very limited	Quite limited	Moderately limited	Somewhat limited	Hardly limited	Not limited

Scaling for answers (points)		1	2	3	4	5	6	7

The questionnaire was devised based on the multi-step procedure [20,21] which is summarized in the study design. The first step was to identify needs and define the operational objectives [22,23]. The questionnaire was developed from an initial inventory of questions and a qualitative study. Cognitive debriefing interviews were conducted to assess respondents' comprehension of questions and response scales. Psychometric testing of QLCCDQ was performed with a group of parents of children with diabetes or asthma. The questionnaires were distributed in a hospital outpatient department, were self - completed by parents and returned to study physicians.

Participants of the study were chosen based on convenience samples. Approval from an ethics committee at the authors' university was obtained by the study center.

### Study design

Questionnaire development was based on a multi-stage procedure which included the processes of item identification, questionnaire development, and psychometric testing:

Process of item identification:

- Semi structured interview with health professionals and parents of children with chronic disease (diabetes, asthma, and eczema)

- identification of most the troublesome problems

- initial inventory development with a 5 point Likert scale rating the importance of each item

- qualitative study among parents of children with chronic disease

Process of questionnaire development:

- questionnaire development based on results of qualitative study

- cognitive debriefing (to assess the comprehension of the questionnaire)

- development of the final version of the questionnaire

- cognitive debriefing of the final version of the questionnaire

The last phase of the study was psychometric testing.

- The validation process included internal consistency, construct validity, reproducibility, and known- group analysis (children with asthma and diabetes)

### Description of process

Semi structured interviews and a literature review were used to develop an initial inventory listing the most troublesome problems for parents of children with chronic disease. Health professionals (pediatricians, nurses) and parents of children with diabetes, asthma or eczema were invited for an interview to help develop this inventory. In the qualitative study which followed, parents were asked to choose from the inventory, which concerns were relevant to their situation and rate the importance of several troublesome issues (from 1 - not important/troublesome to 5 very important/troublesome). Not all parents rated all the items. Inclusion criteria for participation in this phase of the questionnaire development required the child to have a clinical diagnosis of asthma (with possible perennial rhinitis as concomitant disease) or eczema or diabetes; symptoms presence or the diagnose was established at least 12 months before the study; no other social and psychological problems which may have an impact on family functioning or ability to respond (such alcohol/drug abuse, psychiatric diseases), and; willingness to provide consent to participate.

In the next phase, cognitive debriefing interviews were conducted involving parents recruited from the qualitative study. Cognitive debriefing was done twice - first on the primary version and second on the revised questionnaire to ensure that respondents could understand and complete the questionnaire.

The psychometric testing phase to validate the questionnaire consisted of measuring internal consistency, construct validity, reproducibility, and known - group analysis. Psychometric testing was conducted in outpatient departments involving parents of children with asthma or diabetes. Asthma children represented a wide range of severity: episodic, mild and moderate. Children with diabetes were insulin - dependent with or without complications. Both asthma and diabetes study groups included patients with and without satisfactory disease control. Subjects were excluded if there were significant co-morbidities in the child that could impact their quality of life and other social and psychological problems which could have an impact on family functioning or ability to respond. Once completed, the questionnaires were returned to study physicians.

Comparison of HRQOL results in parents whose child was assessed as stable and parents with uncontrolled asthma or diabetes were conducted for both groups. Asthma control is defined based on GINA guidelines as daytime symptoms present less than twice a week, rescue medication use less than twice a week, no nocturnal symptoms, no limitations in physical activity, normal or near normal lung function values in lung function test [24].

Diabetes control was based on glucose and hemoglobin A1c (Hb A1c) levels. Patients were classified as stable if Hb A1c below 6.5% was achieved, as well as glucose if fasted and between meals ranged between 3.9 and 6.1 mmol/l, after meals less than 8.9 mmol/l [25].

The control group for the psychometric testing consisted of a volunteer group of parents of healthy children. The final version of QLCCDQ was completed. As typical childhood diseases or even prophylaxis (vaccinations) could focus parental attention on health - related problems it was important to find out if all questions included in the questionnaire were clearly related to chronic disease conditions. For this reason, we included a control group.

### Statistical analysis

The statistical analysis was performed using standard procedures available in the Statistica 7.1 package (StatSoft Inc, USA) and SAS, version 9.2 (SAS Institute Inc., Gary, NC). Normality of distributions of continuous variables was assessed by the Shapiro-Wilk test. Statistical significance of differences between continuous variables was analyzed by the Student's t-test and Analysis of Variance. If a non-normal distribution was found, the Mann-Whitney U test and Kruskal-Wallis tests were used, respectively. Differences between categorical variables were examined by the Chi-square test. Correlations between variables with non-normal distributions were measured by Spearman's rank correlation coefficient. The reliability of the defined subscales was measured by the Cronbach's alpha. The statistical inferences were based on the level of significance of p < 0.05.

## Results

Semi structured interviews were conducted on the group of health professionals (18 pediatricians, 10 nurses) and 22 parents of children with diabetes, asthma or eczema. Health care professionals raised the emotional context of disease, distress and anxiety, potential limitation of occupational roles as troublesome areas for parents. Parents identified emotional distress, time limited for other family members and professional career, and decreased social interactions as troublesome areas. All important issues raised by respondents were incorporated into the initial inventory, which was distributed during qualitative study.

### Qualitative study

The qualitative study was conducted among 65 parents of children aged 6-14 years old, mean age 9.23 (SD 2.06), with chronic disease including asthma, eczema or diabetes [Table 2].

**Table 2:** Qualitative study - demographic data

	Diabetes	Asthma	Asthma and rhinitis	Eczema
	n = 22	n = 26	n = 3	n = 14
**Parent's gender**				
Male	8	7	1	4
Female	14	19	2	10

**Child's gender**				
Male	7	8	2	5
Female	15	18	1	9

**Child's age**				
Mean (SD)	9.4 (2.1)	8.8 (1.9)	8.0 (2.0)	9.8 (2.3)
Min-Max	6-13	6-14	6-10	6-13

Based on the qualitative study, the most troublesome issues related to the child's disease as identified by parents were emotional distress and feeling depressed, avoiding social interactions, and time limited for other family members. Other concerns reported by parents reflected limitations in occupational activity/professional career and social activities.

Among those parents who rated the concern as relevant, the highest means were for experience anxiety due to child's chronic disease, worried or concerned about child's future, and limitations in social activities due to their disease [Table 3]. We did not find statistically significant differences in the responses between mothers and fathers.

**Table 3:** Reported importance of parent's concerns due to child's chronic disease

Parents' reported concerns	**Percent of respondents**^**1)**^	**Mean scores **^**2)**^	SD	Min	Max
Experience anxiety due to child's chronic disease	100	4.9	0.2	4	5
Worried or concerned about child's future, due to their disease	100	4.9	0.2	4	5
Giving up meeting friends because of child's disease	100	4.7	0.5	4	5
Feeling depressed because of child's disease	100	4.8	0.4	4	5
Experience anxiety due to child's disease/symptoms	100	4.8	0.4	4	5
Struggling to find time to spend with other family members (spouse, another child) because of child's disease	95.4	4.7	0.5	4	5
Limitation in attention given to other family members	95.4	4.7	0.5	4	5
Feeling guilty due to child's disease	7.7	4.6	1.1	3	5
Impact of diesase on own or family's financial situation	27.7	3.5	1.1	2	5
Limitations in work related activities	75.4	4.5	0.5	4	5
Refrained from hobbies/entertainment because of child's disease	86.1	4.6	0.5	4	5
Limitations in household activities (housework, shopping, cleaning)	87.7	4.7	0.5	4	5
Feeling shame as the result of child's disease	3.1	5	0.0	5	5

^1) ^percentage of parents who rated the concern as relevant

^2) ^5-point scales used (1:not important to 5:very important)

### Cognitive debriefing

Cognitive debriefing involved 10 parents of children with diabetes and 15 parents of children with asthma. The debriefing resulted in three questions being removed and the wording of five questions being changed. After making the final version all questions were assessed as viable, relevant to parental concerns about their child's disease, and easy to understand.

### Psychometric testing

Parents of 98 children aged 5-14 years old [mean age 9.07 (SD 2.08)] with asthma or diabetes [Table 4] completed the questionnaire. The control group consisted of 21 parents of healthy children. There were no significant differences in the child's age between the asthma, diabetes groups, and control groups.

**Table 4:** Psychometric testing - demographic data

	Diabetes n = 31	Asthma n = 67	Stable patients n = 73	Unstable patients n = 25	Controls n = 21
**Parent's gender**					
Male	13	22	19	8	9
Female	18	45	54	17	12

**Child's gender**					
Male	16	40	62	13	11
Female	15	27	11	12	10

**Child's age**					
Mean (SD)	9.2 (2.7)	8.5 (1.3)	8.6 (1.6)	9.3 (2.5)	9.0 (1.9)
Min-Max	5-13	7-12	5-13	5-14	5-13

### Internal consistency reliability

The reliability of the defined subscales was evaluated by Cronbach's α. All subscales that were supposed to represent a single - construct scale achieved Cronbach's alpha at least 0.7. The values for subscales ranged between 0.77 and 0.93 [Table 5] thus suggesting acceptable internal consistency of the questionnaire.

**Table 5:** QLCCDQ subscales reliability

Domain	Cronbach's α
Family roles	0.77
Social roles	0.79
Occupational roles	0.91
Roles limitations	0.91
Symptoms	0.93
Emotions	0.91

### Construct validity

Statistically important positive correlations were observed between all QLCCDQ dimensions defined by questionnaire's subscales [Table 6]. Better (higher scores) of defined subscales correlated positively with other subscales as expected; the Spearman coefficients ranged from 0.4 to 0.9. As well, positive correlations were found between most questions representing each subscale [Table 7].

**Table 6:** QLCCDQ subscales Spearmans' correlations

	Family roles	Social roles	Occupational roles	Roles limitations	Symptoms perception	Emotions
**Domain** **QLCCDQ**						
Family roles	-					
Social roles	***0.7*** ***(0.0000001)***	-				
Occupational Roles	***0.7*** ***(0.0000001)***	***0.7*** ***(0.0000001)***	-			
Roles limitations	***0.8*** ***(0.0000001)***	***0.9*** ***(0.0000001)***	***0.9*** ***(0.0000001)***	-		
Symptoms perception	***0.5*** ***(0.00003)***	***0.4*** ***(0.002)***	***0.4*** ***(0.0007)***	***0.5*** ***(0.00003)***	-	
Emotions	***0.7*** ***(0.0000001)***	***0.6*** ***(0.0000001)***	***0.6*** ***(0.00001)***	***0.7*** ***(0.0000001)***	***0.9*** ***(0.0000001)***	-

p-value < 0.05 in brackets (significant in bold)

**Table 7:** Spearman's correlations between questions representing subscales

QLCCDQ subscale	Questions				
		6	13		
			
Family roles	6	-			
			
	13	**0.62546** **(< .0001)**	-		
			
					
		1	2	14	
		
Social roles	1	-			
		
	2	**0.79839** **(< .0001)**	-		
		
	14	**0.62139** **(< .0001)**	**0.72042** **(< .0001)**	-	
					
		10	12	15	
		
Occupational Roles	10	-			
		
	12	**0.38899** **(0.0040)**	-		
		
	15	**0.63701** **(< .0001)**	0.26247 (0.0576)	-	
					
		4	5	8	
		
Symptoms perception	4	-			
		
	5	**0.67511** **(< .0001)**	-		
		
	8	**0.68810** **(< .0001)**	**0.66283** **(< .0001)**	-	
					
		3	7	9	11
	
Emotions	3	-			
	
	7	**0.35271** **(0.0096)**	-		
	
	9	**0.33649** **(0.0138)**	**0.58539** **(< .0001)**	-	
	
	11	**0.30330** **(0.0273)**	**0.43833** **(0.0010)**	**0.47349** **(0.0003)**	-

p-value < 0.05 in brackets (significant in bold)

### Reproducibility

Reproducibility of answers was examined in a subgroup of 22 parents of asthma patients. The questionnaire was distributed twice - parents were asked to complete the questionnaire again 2 hours after first completion. According to the values of weighted kappa coefficients the level of agreement was almost perfect in case of 8 questions (0.81-1.00), substantial for 5 questions (0.61-0.80) and moderate for 2 questions (0.41-0.60) [Table 8].

**Table 8:** Weighted kappa for QLCCDQ questions

Question nr	Weighted kappa	95% CI
1	0.6	0.2; 0.9
2	0.8	0.6; 0.9
3	0.9	0.8; 1.0
4	0.9	0.8; 1.0
5	0.9	0.8; 1.0
6	0.8	0.7; 1.0
7	1.0	1.0; 1.0
8	0.9	0.9; 1.0
9	0.6	0.3; 1.0
10	0.9	0.8; 1.0
11	0.6	0.2; 1.0
12	0.8	0.6; 1.0
13	0.6	0.2; 1.0
14	0.8	0.3; 1.0
15	0.8	0.6; 1.0

p < 0.01

### Known-groups analysis

The discriminative validity was determined by comparing mean scores between parents of children with conditions and parents of control children as well as between parents of children with stable conditions and those with unstable conditions. Higher HRQOL scores were found in parents of control children than parents of children with a chronic condition [Table 9]. Emotions and symptoms domains were most impaired in the chronic disease group. The analysis also revealed that QLCCDQ scores were significantly higher in the group of parents of stable children in comparison with unstable children. Only one subscale didn't show any significant differences - occupational roles functioning [Table 9].

**Table 9:** QLCCDQ scores

Domain	Stable		Unstable		p-value	Chronic disease		Controls		p-value
				
	Mean	SD	Mean	SD		Mean	SD	Mean	SD	
Family roles	5.6	1.2	4.6	1.4	**0.03**	5.2	1.3	6.3	0.9	**0.0004**
Social roles	5.8	1.0	4.9	1.6	**0.01**	5.5	0.6	6.6	1.3	**0.00002**
Occupational	5.4	1.4	4.7	1.8	> 0.5	5.1	0.8	6.4	1.7	**0.0001**
Roles limitations	5.5	1.1	4.7	1.5	**0.04**	5.3	0.7	6.5	1.4	**0.00002**
Emotions	4.5	1.1	3.8	1.1	**0.001**	4.3	1.0	6.2	1.0	**0.0000001**
Symptoms	3.6	1.4	2.8	1.1	**0.003**	3.3	1.3	5.9	1.1	**0.0000001**

p-value < 0.05 (significant in bold)

At this stage, the analysis revealed, that QLCCDQ results showed that mother's scores were lower in comparison with father's scores in most subscales [Table 10].

**Table 10:** Parents' gender and QLCCDQ scores

Domain	Male		Female		p-value
	Mean	SD	Mean	SD	
Family roles	5.61	1.23	4.79	1.42	**0.03**
Social roles	5.45	1.35	4.47	1.71	**0.04**
Occupational	5.38	1.28	4.74	1.68	> 0.5
Roles limitations	5.45	1.17	4.65	1.45	> 0.5
Emotions	5.78	1.05	4.76	1.35	**0.003**
Symptoms	3.74	1.24	2.85	1.30	**0.09**

p-value < 0.05 (significant in bold)

## Discussion

As chronic child's disease may have an impact on the entire family functioning - parents of children representing common chronic diseases in childhood were invited to participate in a qualitative study to identify their most troublesome concerns. Most parents identified emotional concerns and impact on roles functioning as primary concerns. Parents complained about their limitation in attention and time offered to other family members, limitation in work related activities, as well as daily house-hold activities. Based on the qualitative study, the questionnaire was developed and cognitive debriefing was subsequently used to generate the final version of the new tool.

This study investigated the validity of newly developed questionnaire addressing the HRQOL of parents of children with a chronic condition. Psychometric analyses based on a sample of 65 parents of children with asthma or diabetes was used to evaluate the validity of the questionnaire. With the satisfactory results of the QLCCDQ found in this study, our results would suggest that this questionnaire could be used in clinical practice. Psychometric properties for the questionnaire including internal consistency, construct validity and reproducibility were satisfactory. Internal consistency of QLCCDQ scores achieved acceptable levels of Cronbach' alpha. The QLCCDQ demonstrated good reproducibility with kappa coefficients of at least substantial for most questions.

Construct validity of the questionnaire demonstrated positive correlations between all defined subscales. Parents of children with chronic disease demonstrated lower HRQOL mean scores in all subscales in comparison to parents of control children. The comparison between QLCCDQ scores of parents of stable children and unstable children has shown that clinical status had an impact on parental functioning. Significantly lower QLCCDQ scores have been observed for most subscales for parents of children with unstable conditions. The only exception was the occupational roles subscale where no differences were found between parents of stable and unstable children.

The initial inventory didn't show that parental concerns due to a child' disease differed between mothers and fathers. Thus HRQOL questionnaire should not include different items for mothers and fathers. As it was found in other studies our analysis of QLCCDQ results showed that the HRQOL due to child's disease was more impaired in mothers [26].

Our study showed that the QLCCDQ could bring new insights into health related outcomes in childhood diseases. The questionnaire showed that HRQOL scores in parents were statistically lower in the case of chronic disease in comparison with parents of healthy children for all subscales. It is worthwhile noting that answers to only one question, feeling guilty due to the child's disease, didn't showed any differences between the control and the chronic condition group in the psychometric testing. Despite of qualitative study results showing that guilty feeling as the result of child's disease were found as an important concern, no differences were found between scores of controls and chronic conditions. This may be important when using the QLCCDQ in other childhood chronic diseases as the child's disease may have an impact on contexts such as emotional functioning.

Parental HRQOL assessment using QLCCDQ could be beneficial in clinical practice. The implementation of the tool with parents of children with asthma or diabetes was successful and would be helpful in team-oriented approaches in patient care, where parents would become integral part of the team involved in therapy.

QLCCDQ has a potential to contribute to many aspects of routine care including improved detection of emotional consequences of child's disease and assessment of treatment outcomes in the context of family functioning. The HRQOL feedback can provide important information for both child and family tailored approach in treatment. Studying the feasibility of this questionnaire to measure HRQOL of parents of children with other chronic conditions as the result of child' s disease would be of great interest and subject to further research.

## Conclusions

The QLCCDQ shows good internal consistency, test-retest reliability, and construct validity. The questionnaire may be useful in helping to understand the impact chronic child's disease on parental perception of health outcomes.

## List of abbreviations

CHQ PF 50: Child Health Outcomes Questionnaire Parent Form; HRQOL: health - related quality of life; PACQLQ: Pediatric Asthma Caregiver's Quality of Life Questionnaire; PAQLQ: Pediatric Asthma Quality of Life Questionnaire; PDQOL: Pediatric Diabetes Quality of Life Questionnaire; QLCCDQ: Quality of Life in a Child's Chronic Disease Questionnaire; SF 36: Medical Outcomes Survey Short Form; WE-CARE: Well being and Satisfaction of CAREgivers for Children with Diabetes Questionnaire.

## Competing interests

The authors declare that they have no competing interests.

## Authors' contributions

MF, JZ, WP and GB conceived of the study and participated in study design, data interpretation. ŁW MF conducted qualitative study, cognitive debriefing and developed the questionnaire, MF, GB, MS JZ WP conducted psychometric study, GB, ŁW, MS were responsible for statistical analysis. MF, GB, ŁW worked on manuscript. All authors read and approved the final manuscript.
